# Annual Report of the Section on Obstetrics and Diseases of Women and Children of the Chicago Society of Physicians and Surgeons

**Published:** 1874-02

**Authors:** A. Reeves Jackson

**Affiliations:** Chairman of the Section


ARTICLE II.—*Annual Report of the Section on Obstetrics and Diseases of Women and Children of the Chicago Society of Physicians and Surgeons. Department of Gynæcology.* Read before the Society December 8, 1873, by A. REEVES JACKSON, M.D., Chairman of the Section.

In preparing the report upon gynæcology, which I have the honor to submit to the Society, I have been governed by one principal object. This was, to bring together the most recent expression of the opinions held by prominent gynæcologists, in different parts of the world, upon various important subjects connected with this department of practical medicine. I hoped, in this way, to present the subject in such a light as would enable the Society to appreciate the progress and present status of gynæcological doctrine and practice.

The field from which my material has been culled, consists of such of the various medical periodicals published during the current year as I have been able to consult. The report, therefore, is simply a compilation. In no case have I ventured to intrude my own opinions upon the subjects discussed; preferring to state facts and views as given by others, and permitting the members of the Society to form their own conclusions.

I may premise, however, that in gynæcology there is no more uniformity of belief, as regards questions of pathology or treatment, than is found in other branches of medical practice.

In many instances the diversity of opinion amounts to complete opposition; and, while not attaining this extent in others, it is yet so great as to make them irreconcilable. One set of practitioners, for example, advocate the use of mechanical appliances as the chief and sufficient means for the treatment of uterine displacements; others decry their use as unnecessary and hurtful, and condemn them *in toto*. One set, again, believing that all, or nearly all, cases of painful menstruation depend upon mechanical obstruction to the flow, tell us that the uterine cavity must be dilated; and they differ as to whether we will accomplish this end best by bougie, sponge or sea-tangle. Others, who agree with these last as to the pathological condition present, differ from them in advising that the uterine canal be not stretched at all, but cut; and then they, too, differ among themselves as to where the cutting shall be done—whether at the internal or external os uteri—and whether it shall be done with one blade or two, bilaterally or antero-posteriorly.

And so, were it profitable and not wearisome, I might go on and show that upon almost every point there is sufficient variance of opinion to prove that gynæcology is a human science; and being human, imperfect; and being imperfect, susceptible of improvement.

### TREATMENT OF UTERINE CANCER BY ERGOT AND ESCHAROTICS.

Dr. Alexander Milne, in a paper read before the Obstetrical Society of Edinburgh, and an abstract of which was published in the *Obstetrical Journal of Great Britain and Ireland*, for May, 1873, advocates the use of caustics, on the ground that, in addition to their corrosive properties, they possess also an alterative and eclectic influence; that they not only sever the morbid part, but promote a healthy cicatrix behind; and furthermore, that they penetrate and search out and destroy those deeper morbid cells which are removed some distance from the parent tumor, and which insure its recurrence.

He prefers to begin with the dried sulphate of zinc, applied to the cervix pretty freely through the speculum, the vagina being immediately thereafter plugged with cotton wool, tipped at the uterine end with a little olive oil. This is to be applied until a slough comes away, after which the cervix is to be injected with the nitrate of copper. This last is done in order to attack any morbid cells lying beyond the sore from which the slough has separated. He regards the nitrate of copper as being the best adapted to elect, attack and destroy these lurking microscopic cells, which, if allowed to remain, are a sure guarantee of a fresh growth.

In reference to the influence of ergot, given internally, in cancer, Dr. Milne believes that the drug, in addition to its power as a hemostatic, for which it has usually been administered, has also another effect—it leads to the atrophy of the uterus, an observation which he claims to have been the first to make. It acts beneficially by diminishing the afflux of blood to the uterus, thereby combatting uterine congestion—a condition present in malignant disease—and also by its no less important property of inducing uterine atrophy. By the combined use of the escharotics and ergot he has succeeded in curing two cases of cauliflower excrescence, and in three cases of the medullary form of the disease he had at least retarded its progress. In one of these last he thought a permanent cure would be effected, while as regarded the others, there had been diminution of pain, of bleeding, and of offensive discharge.

### UTERINE CANCER—ITS TREATMENT BY GASTRIC JUICE.

Dr. C. H. F. Routh read a paper upon this subject before the Obstetrical Section of the British Medical Association, advocating the use of gastric juice as a dressing to cancerous ulcers of the uterus—not for the purpose of superseding other means of treatment, but only as a powerful adjuvant. Dr. Routh's plan is to assist the healing process, after the whole, or part only, of the diseased mass has been removed. He directs that the cancerous growth about to be treated should have its surface destroyed, if it be not already in a state of ulceration; but even in these latter cases he thinks it well to remove a considerable portion. It matters not by what agent this is done—the knife, the ecraseur, strong caustics, or the actual cautery. He has used all, according to the case. After a raw surface is formed, or even if it be still covered by a slough, which is the result of the caustics or cautery, he applies the gastric juice in excess, on a piece of lint. He does this through a speculum. This is covered with a piece of oiled silk or gutta-percha sheeting, and the whole kept in place by a plug of cotton. This should be done twice a day, or oftener if it be practicable.

Dr. Routh says: "Let it be clearly understood that I am far from saying that gastric juice will cure all cases of cancer. But I am sure it will cure some where other agents have failed."

He then relates four cases in which this plan of treatment had been successfully used, two of them being under his own care, and the other two under the care of other practitioners. The length of time during which these patients were under treatment was, respectively, thirteen, twenty-one, thirty-two and thirty-five days.

### FORCIBLE AND RAPID DILATATION OF THE CERVIX UTERI FOR THE CURE OF DYSMENORRHŒA.

Dr. Ball, in the *New York Medical Journal*, for October, 1873, details nine cases of dysmenorrhœa in which great success attended forcible dilatation of the cervix uteri.

The following is Dr. Ball's method in detail:

"The bowels are thoroughly evacuated, the patient put under the influence of an anæsthetic, the cervix seized with a tenaculum and drawn down, and a metal bougie, as large as the canal will admit, introduced, followed in rapid succession by others of a larger size, until No. 7 is reached, which represents the size of the dilator used by Dr. Ball, which is then introduced, and the cervix stretched, in every direction, until large enough to admit a No. 16 bougie, which is all that is generally necessary. A hollow gum-elastic uterine pessary, of about that size, is then introduced and retained in position by a stem secured outside the vulva, which is allowed to remain about a week. During this time the patient is kept in bed, usually on her back.

"The effects of this operation are three-fold: first, by breaking up all adhesions, which are often very firm and unyielding, it relieves the constriction entirely, while, acting as a derivative, it cures the hyperemia of the cervix; and further, it establishes a radical change in the nutrition of the whole organ. . . . . .

"In cases of flexion, the relief is obtained by the straightening of the canal, which is produced by a change of the muscular tissue of the cervix from an abnormal to a normal condition. In the rapid dilatation of the parts, the constricting fibres are, of course, lacerated to some extent, and in healing up around the pessary must, necessarily, conform to their new relation."

Dr. Ball regards this method of treatment as having one great advantage, viz., the saving of time, and alleges that he has accomplished more by it in a less number of weeks than it would take months to do by the ordinary method. He thinks it produces less constitutional disturbance than tents, and is safer than the metrotome, and free from some of the objections to the latter; as, for instance, when incisions are made through the tissues of the cervix, unless carried deep enough to prevent reunion, they must, of necessity, form a cicatrix, which will interfere more or less with the dilatation of the parts; while, if the operation does not succeed, the patient is left in a worse condition than before.

### HYSTEROTOMY *versus* DILATATION.

On the other hand, Dr. Percy Boulton, in a paper read before the Harvein Society, March 6th, of which an abstract is given in the *Obstetrical Journal of Great Britain and Ireland*, on the subject of Hysterotomy *versus* Dilatation, announces his preference for the former over any form of dilatation.

The condition calling for the operation is the so-called mechanical dysmenorrhœa. These cases are due to a preternatural narrowing of one of the orifices of the uterus or the cervical canal, with or without flexion, and generally associated with ovarian pain and other symptoms. This stricture might be congenital, or the result of strong caustics, applied for the cure of ulceration or other purpose. Every one is agreed that the only thing to be done is to enlarge the passage through which the menstrual discharge has to flow, and one author thinks that if this is not done there is great danger that the constantly recurring congestion and irritation will eventually produce ovarian disease.

For the cutting part of the operation, he advocates the use of the metrotome *cache*. 1. Because the part introduced into the uterus is smaller than any other instrument used for the purpose, and is passed more easily. 2. It requires only once introducing, on account of cutting bilaterally, and therefore causes less irritation. 3. There is little or no danger of wounding the circular arteries of the os internum, or the vagina, in withdrawing the instrument. 4. It can be used without a speculum.

After the cutting is over, Dr. Boulton prefers a stem—not a plug—to any other dressing.

The author then gives the results of one hundred cases of hysterotomy, operated upon by Dr. Thomas Hawkes Tanner, or himself, five hundred by Drs. Sims and Emmet, and three hundred by Dr. Greenhalgh—nine hundred in all, and shows that in this number hemorrhage, requiring plugging, occurred in two, pelvic cellulitis in six, and in one, fatal peritonitis was set up; but the patient having had tubercular peritonitis before operation, this was an unsuitable case. This was the only case of death.

Dr. Boulton then asks: "Would such evidence be considered favorable or not in any other operation? Is there any other operation which gives equal relief to the patient which is equally free from danger?"

He calls attention to the fact that dilatation of the os uteri takes place at every confinement, and whenever any intra-uterine tumor is removed; but as certainly as the uterus is left in repose so certainly does it close again. How could any one expect, then, he asks, any permanent cure from dilatation? He prefers hysterotomy because, after it the cure is generally perfect and permanent, provided the stem is worn at least four weeks, while a cure is rarely permanent after dilatation; and because cutting gives relief rather than being a source of irritation, while the reverse is true of dilatation.

In conclusion, he details six cases in which he had cured both dysmenorrhœa and sterility by hysterotomy, in two of which dilatation had been tried without success.

TREATMENT OF DYSMENORRHŒA.

Dr. Charles R. Drysdale, in the *Medical Press and Circular*, for October, has a paper upon this subject, in which he differs from both the preceding authors. After detailing the treatment useful for the neuralgic and congestive forms of the disease, he expresses the opinion that the cases of painful menstruation depending upon mechanical stoppage to the flow are comparatively rare; and that the assertion of Dr. Marion Sims that the treatment of the majority of uterine diseases should be surgical, seems to the author absurd in the highest degree. He further states that, according to the experience and statements of Drs. Kidd, of Dublin, and Gream, of London, the operation of incising the cervix, which is advised even more by Dr. Sims than by Dr. Simpson or Spencer Wells, may produce fatal hemorrhage, pelvic abscess, a consecutive relaxation which is prejudicial to gestation, or a cicatrix. He also refers to the diverse practice of Drs. Barnes, Greenhalgh and Routh; the former, in cases of conical cervix, dividing the external os uteri, while the two latter say that in the great majority of cases the stricture is at the internal os uteri.

Dr. Drysdale advises, in cases of mechanical dysmenorrhœa, the introduction of various sizes of sounds, or the use of tents of laminaria digitata, until the uterus is large enough to admit a sound of the size of a No. 9 catheter. He considers the hysterotome as only required in cases of cicatrix after laborious confinements.

### SUBINVOLUTION OF THE WOMB.

In the *Charlestown Medical Journal*, for May and September, there appeared articles upon the above subject by Dr. Baruch, in which the author calls attention to the improper diet of lying-in women, which he regards as a frequent etiological element in the production of the abnormal condition. He says: "They are fed during the first week on tea, toast and gruel; all stimulating and sustaining articles of diet are sedulously avoided, lest their 'heating properties' produce inflammation. The patient's stamina is impaired, the circulation suffers, and the due execution of the local processes of degeneration and absorption are interrupted."

Dr. Baruch recommends as measures of treatment the introduction of Simpson's large sound for a few minutes, twice a week, with the administration of full doses of fluid extract of ergot. Should these measures fail, the weekly introduction of sponge-tents should be tried. When the cervix has become indurated, hot water douches should be used, the Fountain Syringe being the best instrument for the purpose. Should erosions be found on the cervix, they should be touched once a week, after being thoroughly cleansed of the mucus or pus which envelops them, with chromic acid, one part to three of water, or the pure tinct. iodine. The application should be followed by the introduction of a plug of cotton, saturated with glycerine, which should be retained twelve hours. If the erosions extend into the uterus, weak solutions of iodine, chromic acid, sulphate of zinc or carbolic acid may be introduced by bits of sponge, held in Sims'

sponge-holder. Counter-irritation to the lumbar region, with hip-baths, are also useful.

NITRIC ACID IN THE TREATMENT OF UTERINE DISEASE.

Dr. Lombe Atthill, in a paper contributed to the *Obstetrical Journal of Great Britain and Ireland*, for June, 1873, relates several cases in which he had used the fuming nitric acid in the treatment of various chronic forms of uterine disease.

The cases treated were menorrhagia, connected with a rough granular condition of the uterine lining membrane, subinvolution, endometritis, and for checking the hemorrhage dependent upon imbedded fibrous tumors. As the result of Dr. Athill's experience, he draws the following conclusions:

1. Where tenderness on pressure exists, it should be removed, or at least materially lessened, by local depletion, before the acid is used.

2. That when this precaution has been taken, fuming nitric acid may be applied with safety to the interior of the uterus.

3. That when the cervix has been previously dilated, its application does not cause any pain.

4. That in some instances it appears to have a directly soothing influence on the uterine nerves.

5. That when applied through a canula, pain is sometimes produced, but less severe in character than that caused by the use of the solid nitrate of silver.

6. That its use is in some cases followed by hemorrhage of moderate amount, which, however, does not influence the result of the case.

7. That if applied to the *healthy* cervix, it may produce contraction, and possibly obliteration of the cervical canal, and that consequently, means should be adopted to guard the cervical canal, when *healthy*, from its action.

8. That in cases where imbedded fibrous tumors exist, the fuming nitric acid exercises a marked effect in controlling hemorrhage and in allaying pain.

Dr. Atthill's mode of applying the acid is as follows: Having previously dilated the cervical canal by means of sea-tangle tents, he seizes the anterior lip of the cervix by means of a vulsellum, and thus draws down and steadies the uterus; he then introduces a bivalve intra-uterine speculum, made of vulcanite, to the depth of about an inch, and expands the blades slowly to an extent sufficient to permit a pair of fine forceps, holding a roll of cotton, to be introduced. With the latter he dries the inner surface of the uterus, and withdrawing the cotton, passes through the speculum a probe armed with a roll of cotton saturated with the fuming nitric acid. This being then withdrawn, the blades of the speculum are closed. A pledget of cotton soaked in glycerine is placed in the vagina, a strong thread being attached to facilitate removal, the lip freed from the grasp of the vulsellum, and the patient put to bed and kept quiet for some days.

### ON INTRA-UTERINE MEDICATION.

Dr. Robert Barnes, in the *British Medical Journal* for January 11, has a paper upon the different forms of intra-uterine treatment. Speaking of intra-uterine injections, he says, several precepts have been enjoined to avoid their dangers. The great object aimed at is to avoid or lessen the risk of the fluid running along the Fallopian tubes. This is sought to be attained—1. By securing free dilatation of the cervix uteri before injecting, so that the fluid may readily run back into the vagina. For this purpose the preliminary use of laminaria tents is advised. 2. By using only graduated quantities of fluids, and injecting very gently and slowly. 3. By using a double canula, so as to secure a return current. To effect this the more surely, the openings of the canula at the uterine end are made at different levels.

Dr. Barnes has not much faith in the double canula. The end which should serve for the return current is liable to be choked. The preliminary free dilatation of the cervix, and the use of gentleness in propelling the fluid, should never be omitted; but the author does not believe that the observance of these precautions is an absolute guaranty against accidents. He thinks it better to use injecting-pipes having lateral openings of very fine calibre, so as to "pulverize" the liquid.

Dr. Barnes strongly advises not to use injections at all in cases of marked flexion of the uterus. Even if we dilate the cervix first by tents, and maintain the uterus erect during the injection, we cannot always be sure that the flexion will not be reproduced, so as to prevent the issue of the fluid; and it must not be forgotten that it is especially in these cases that the uterine cavity is likely to be enlarged, and the Fallopian tubes dilated. For all these reasons, Dr. Barnes would restrict the use of intra-uterine injections within the narrowest limits. He rarely employs them now, except in cases of urgent danger from menorrhagia.

Besides, we may obtain almost all the advantages that injections are capable of giving by other means. For example, the agents which are useful in the form of solutions for injections, may be employed by swabbing, or solid, or in the form of ointment. Thus, where the use of chromic or nitric acid, or perchloride of iron, or iodine, or bromine, is indicated, these agents can be applied soaked on a sponge or piece of cotton, or a glass or hair pencil, the cervix having been previously well dilated. Nitrate of silver is best applied in the solid form. To lessen its liability to cause severe uterine colic, it should be fused with an equal quantity of nitrate of potash. Dr. Barnes objects to the ordinary way of using the solid nitrate of silver—that is, by holding a piece of the stick in a forceps or *porte-caustique*. The piece may fall out or break, and a fragment left behind in the cervix or body of the uterus may give rise to intense agony, and even metritis. To avoid this, Dr. Barnes adopts a contrivance which he learned from Sir Benjamin Brodie, who armed the ordinary probe by dipping the end into nitrate of silver fused in a watch-glass over a spirit-lamp. The author uses probes of platinum or silver, mounted on handles of convenient length. These probes may be curved to follow the course of the uterine canal. This, Dr. Barnes alleges, is by far the best way of applying nitrate of silver to the os and cervix uteri, and the only safe way of applying it to the interior of the uterine cavity.

One of the most widely useful topical applications to the mucous membrane of the cervix and body of the uterus is sulphate of zinc. It should be fused into small cylindrical sticks weighing three and five grains. These can be carried quite into the uterus without having touched the vagina by the way, by the use of a canula. This should be of the size of a No. 8 or 9 catheter, gently curved, open at the end, and supplied with a stilet or piston. The stick of sulphate of zinc is placed in the uterine end of the canula; the instrument is then passed into the uterus, the patient lying on her left side; and the operator's finger, placed on the os uteri, guides the instrument. This may be done without the use of the speculum. When the instrument has gone the proper depth, the piston pushes out the stick, and the instrument is withdrawn, leaving the stick to dissolve. This it soon begins to do, and by its speedy effect in constringing the mucous membrane, it keeps itself *in situ* until it is completely dissolved.

A most precious way of applying astringents, caustics, solvents or alteratives to the interior of the uterus, is in the form of ointment or pasma. In this form we may use substances which cannot easily be applied in any other way. For example, we can hardly use bromine, or iodine, or mercury, in a solid shape, and to use them in liquid form is open to the objections already discussed. Almost anything can be made into an ointment or pasma, and thus we get a complete practical command over a large range of useful substances.

To introduce an ointment into the cavity of the uterus, Dr. Barnes uses an instrument similar to the one just described for using the sticks of sulphate of zinc, and capable, like that, of being used like a sound without the speculum. The instrument is charged by dipping it into the ointment, a sufficient quantity of which is carried into the uterus, and, by pushing up the piston, is deposited there.

If it be desired to apply a powerful liquid caustic, as chromic acid, or strong bromine, to the interior of the uterus, this can be done by the caustic-carrier. A few shreds of asbestos may be packed in the space between the eyelet-holes and charged with the fluid. On ramming down the piston, the fluid will be squeezed out.

PROLAPSUS UTERI.

Dr. G. P. Hochenberg reports in the *New York Medical Record* several cases of prolapsus uteri in which he found the use of tannin successful when other means had failed to give relief. His method is as follows: A glass speculum is introduced into the vagina, so as to push the uterus into its place. Through the speculum a metallic tube or syringe, with the end containing about thirty grains of tannin, is passed. With a piston the tannin is now pushed against the uterus, the syringe withdrawn, and the packing neatly and effectually completed with a dry probang, around the mouth and neck of the womb. After the packing is completed, the probang is placed against the tannin, in order to hold it, and the speculum is partially withdrawn. The packing is now fully secured, and the instrument removed.

The application of tannin holds the uterus firmly and securely in place, not by dilatation of the walls of the vagina, but by corrugating and contracting its parts. At first the application may be made weekly, finally but once or twice a month.

Dr. Evory Kennedy, in the *British Medical Journal*, of August 17, reports a case of obstinate procidentia in a widow, aged sixty, who had borne six children, and who could not retain any form of pessary, in which he effected a cure by the use of the actual cautery. The iron was applied about an inch and a half from the vulva, round the surface of the vagina, to the extent of about half an inch. The subsequent treatment consisted of the application of the nitrate of silver. At the expiration of two months she was able to go about again. The uterus remained *in situ*, the cicatrix formed by the eschar preventing the descent of the organ.

Finally, Dr. Chas. R. Drysdale, in the *Medical Press and Circular* of October 23, in a paper upon this subject, advises that in cases of prolapsus, attended with inflammation and enlargement of the uterus, these conditions should be removed before having recourse to any other surgical procedure. But in all cases where there is rupture of the perineum (the uterus being of normal bulk), in all cases of procidentia, when great suffering results from prolapse, when the case is chronic, or when the bladder or rectum is much prolapsed, supports are clearly indicated. Nothing is of more importance than the choice of a pessary in the treatment of any form of prolapse of the uterus. The air-ball pessary of Gariel, or the air-ring, is often useful. The author dislikes Zwauke's pessary; Hodges' horse-shoe pessary is useful, but he prefers the simple ring pessary introduced by Dr. Grailly Hewitt, and thinks they are by far the best ever brought into practice. Hewitt's ring pessary is merely a copper wire ring, coated externally with gutta-percha, of various diameters, which can be moulded into any shape the practitioner pleases, thus being suitable for all cases of introversion, retroversion and prolapse. Sponge pessaries should never be used, as they become rapidly most horribly fetid. Stem pessaries are expensive and difficult to manage, and the author cannot recommend any of them. Ashburvei's pad is an admirable support in cases of rupture of the perineum and procidentia. The most important purpose served by all pessaries which prove of any service, is to keep the uterus motionless. Two important points should be observed in the use of pessaries: one is, not to permit them to remain more than a week at a time in the vagina, and the other, that the very smallest size of pessary that will keep the uterus up should alone be used.

THE INTRA-UTERINE STEM IN THE TREATMENT OF FLEXIONS OF THE UTERUS.

There appears to be an increasing tendency among gynæcologists to resort to the use of the intra-uterine stem for the cure of flexions of the uterus.

Dr. Thomas Chambers, Physician to the Chelsea Hospital for Women, reports in the *Obstetrical Journal of Great Britain and Ireland*, several cases successfully treated by this means. Dr. Thomas Savage, Surgeon to the Birmingham Hospital for Women, in the November number of the same journal, states that he has used the intra-uterine stem for retroflections and anteflexions, in forty-four women who had not been impregnated, and in not one had any ill effects followed. The discharge produced by the presence of the instrument was slight, as a rule, but even when profuse had not been found troublesome, and could be kept in check by the frequent use of the ordinary astringent injections. It had always seemed to disappear on the use of the instrument being discontinued. Dr. Savage had tried the galvanic stem as usually sold, also the modification of it with a bulbous extremity, the plain vulcanite stem, Dr. Greenhalgh's stem, one devised by Mr. Ross Jordan, with a perforation near the extremity through which was passed a thread of India-rubber, after the manner of the winged catheters; but the tendency of all was to slip out. Dr. Chambers' stems seemed most likely to remain in without assistance, but in two instances they too had escaped. The padlock of Grailly Hewitt was in some cases an admirable contrivance, and most frequently remained *in situ*. The best means to adopt was to insert a stem, and keep it in place by a shelf pessary cut small, or a small ring elongated, and moulded to the size and shape of the vagina. The supports of Dr. Wynn Williams seemed to be very useful; they consist of an oval ring, the centre being occupied by a perforated diaphragm of gutta-percha.

### FIBROUS TUMORS OF THE UTERUS.

In the *Half-Yearly Abstract of the Medical Sciences*, for January, 1873, there was published a *resume* of a paper originally published in the *Gazette Hebdomadaire*, November 27, 1872, by Dr. Hildebrandt, in which he reports eight cases of fibrous tumor of the womb, treated by the subcutaneous injection of ergotin.

Langenbeck had already practiced injections of ergotin as a means of treating aneurism, and MM. Ruben and Zeute have directed attention to the good effects of the same treatment when applied in cases of menorrhagia and metrorrhagia after delivery. Dr. Hildebrandt has extended the use of this method, and expresses the hope that he has found in it a new means of treating fibrous tumors of the uterus. The solution used by Dr. Hildebrandt was made up of three parts of ergotin, 7.5 parts of glycerine, and 7.5 parts of water, used with a Pravaz's syringe. This solution produces much less pain than that used by Langenbeck, which contained alcohol. Dr. Hildebrandt states that these injections cause very little pain, and do not produce abscesses. The patient, after each injection with this solution, may return to her home. The site selected for the insertion of the syringe, was that part of the integument of the abdomen immediately over the tumor. The author remarks that the lower parts of the abdomen are more sensitive to puncture and injection than the parts around the umbilicus.

Judging from this report, ergotin administered by injection seems to be a powerful medicinal agent. In one of the cases a tumor which had extended beyond the umbilicus had disappeared; in a second, a tumor which reached as far as the false ribs, descended to below the umbilicus; and in four cases, where the treatment had been less complete, there was improvement in both the general and local conditions. The ergotin rendered menstruation less irregular, less abundant, less prolonged, and, above all, less painful. It is not easy to state precisely the mode of action of the ergotin in these cases. It is probable that in consequence of the contractions produced by ergotin in the nutrient vessels of the tumor, and in consequence, also, of the compression in all directions by the contracting uterine walls, the nutrition of the tumor is interfered with, and that then fatty degeneration and absorption take place. It is probable, also, that intra-uterine tumors are more readily modified in this way than subperitoneal tumors, and ungomata more readily than fibromata.

Dr. George H. Kidd also had a communication upon the subject of fibrous tumors, which was read before the Dublin Obstetrical Society, and which was reported in the *Medical Press and Circular* of August 28.

Dr. Kidd recommends, in the case of such tumors as are removable by this means, the use of steel wire, in fact, a piece of piano-string, with the *ecraseur* instead of the soft iron wire commonly employed. The author says that the soft wire when passed up assumes the shape of the canal of the cervix, and it is difficult to expand it again so as to get it around a large tumor. By using a steel wire you can compress it to get it through the narrow os, and when you get it up it expands by its own elasticity, and you can slip it over the tumor with ease.

Dr. Kidd has used this means satisfactorily in several cases, and the procedure has been adopted by Dr. Atthill and other prominent British gynæcologists, all of whom speak in high terms of its great advantages over the method formerly used.

Finally, Dr. Abeille reports, in the *Gazette Medicale de Paris*, a case of successful removal of a large interstitial fibroma of the uterus, and, in his subsequent remarks upon the subject of pedunculated tumors and polypi, states that when the pedicle is attached at the level of, or a little above, the internal os uteri, whatever may be the size of the tumor, a period will certainly arrive at which the uterus, contracting energetically at a menstrual period, causes the growth to protrude from the orifice after more or less considerable hemorrhage. When the tumor has passed into the vagina, provided it is still fixed to the uterus, the flow of blood becomes continuous. This sign indicates the necessity for an early operation. These are simple cases, and the operation, which consists in cutting through the pedicle, is very easy. Even when the pedicle is attached at a higher part of the uterine cavity (even at its middle, the uterus being divided into two portions by a transverse line), the tumor may still be expelled during uterine contractions. However, for this result to take place spontaneously, more time and more energetic contractions of the uterus are required. The nearer the attachment of the tumor is to the fundus, the more difficult does it become for the uterus to expel it from its cavity, notwithstanding energetic and repeated contractions. When the pedicle is inserted directly at the fundus of the womb, whatever be the size and thickness of the pedicle, spontaneous expulsion of the tumor is absolutely impossible.

Serious mistakes in diagnosis have been made by very experienced practitioners in consequence of their exploring the uterus between the menstrual periods. It is of the highest importance to remember that in all cases where the symptoms lead one to suspect the presence of an intra-uterine polypus, the exploration ought to be made during the menstrual flow, because it is during this period that the uterus, endeavoring to expel the tumor, pushes it towards the cervix, and causes great dilatation at this region.

### THE CONSTITUTIONAL TREATMENT OF CHRONIC INFLAMMATION OF THE CERVIX UTERI.

Dr. Thomas More Madden read a paper upon this subject before the Dublin Obstetrical Society, February 8, of which an abstract was reported in the *Medical Press and Circular.*

In this essay, Dr. Madden expresses the opinion that physicians do not sufficiently realize the constitutional causation of the disease, and that this accounts for the vague and unsatisfactory character of the treatment, which is generally protracted, and frequently unrewarded by cure. Among the consequences of chronic inflammation of the cervix, Dr. Madden includes endometritis, ulceration and hypertrophy of the cervix, menorrhagia, vaginitis, leucorrhœa, hysteria, and, above all, sterility. The author thinks that undue importance is attached to the local exciting causes of these affections, while the constitutional predisposing causes are disregarded. He considers the scrofulous diathesis as one of the most common predisposing causes at work, and thinks that if the connection between struma and the more common forms of uterine disease were recognized, it would lead to a more satisfactory and rational mode of treating these complaints.

Dr. Madden, while fully appreciating the importance of local treatment, yet thinks that almost exclusive attention is paid to it, while the constitutional derangement is practically neglected.

As constitutional measures, the author recommends the simple preparations of iodine, given in small doses, as originally ordered by Lugol, such, for example, as the one-eighth of a grain of iodine with one-fourth of a grain of iodide of potassium, in preference to the larger doses of its compounds now generally prescribed. In cases where symptoms of anemia predominate, the various combinations of steel with iodine are of singular benefit, all symptoms of uterine disease often disappearing under their use without any local treatment whatever. He has found no remedy so generally useful in these cases as a mixture of equal parts of cod-liver oil and syrup of iodide of iron. Other constitutional conditions than the scrofulous diathesis may be the predisposing causes of uterine disease, and here a modified antiphlogistic course of treatment is indicated. Frequently small doses of the bichloride of mercury, $\frac{1}{24}$ of a grain three times a day, in the tincture or infusion of bark, will be found useful. Notwithstanding the prevailing scepticism as to the efficacy of medicines in these chronic uterine affections, Dr. Madden's experience of many cases has fully convinced him that, although the local symptoms may subside for a time under purely local treatment, the patient is more quickly as well as more permanently cured by the administration of constitutional remedies, such as those just referred to, whilst due attention is, at the same time, paid to the local treatment of the ulcerated or inflamed part. The prevailing type of chronic uterine complaints, like that of all other general diseases of the present time, is essentially asthenic, and requires the administration of tonics in almost every instance, and more especially the preparations of steel, iodine and quinine combined, when circumstances admit of it, with change of air and mineral waters. Dr. Madden speaks with great confidence of the curative effects of climate and of mineral and thermal waters in cases of chronic uterine disease, having given his attention to the subject during several years of travel and clinical observation in the health resorts of the Continent and the Mediterranean shores of Europe and Africa, as well as at the spas of Germany, France and Italy.

### ON THE USE OF DRAINAGE TUBES FOR THE PREVENTION OF SEPTICEMIA AFTER OVARIOTOMY.

Dr. J. Marion Sims has contributed to the *New York Medical Journal*, for December, 1872, an interesting paper upon ovariotomy, which has since been published in pamphlet form, in which he expresses the belief that as regards the question of the treatment of the pedicle, the clamp has seen its best days, and that when it comes to be positively determined, he has scarcely a doubt that the decision will be against the principle of drawing the pedicle externally; and, although himself and Dr. Emmet have for the past ten years advocated and practiced the plan of tying the pedicle with silver wire and dropping it back into the abdomen, he yet thinks "the time may possibly come when clamps and ligatures, whether of twine, catgut or wire, may give way to torsion of the arteries, or to their obliteration by enucleation of the pedicle from the coats of the cyst."

But the most important portion of Dr. Sims' paper relates to another matter. From a study of his own *post-mortems* and those of Mr. Spencer Wells, he arrives at the conclusion that "septicemia is the great outlet of life in ovariotomy."

In Mr. Spencer Wells' twenty-eight fatal cases in which *post-mortem* examinations were made, two died of tetanus, and of the remaining twenty-six, "two had pyemic poisoning, and twenty-four presented the infallible *post-obit* evidences of septicemia. In every one of Dr. Sims' seven *post-mortem* examinations, and in every one of Mr. Wells' twenty-six, were found uniformly the same pathological appearances. In all of them was found a quantity of reddish or grayish turbia, or fetid, or putrid, or acrimonious serum in the peritoneal cavity; and in cases slowly dying of pyemia, were invariably found pyogenic reservoirs in the pelvic cavity. In view of these facts, Dr. Sims asks, "Is it not logical to infer that these pent-up fluids are the causes of the blood-poisoning that so uniformly, I should say universally, attends fatal cases of ovariotomy?

"I do not pretend to deny that death may occur from shock, or from hemorrhage, or from heart-clot, or from exhaustion; but I feel sure that these, independently of blood-poisoning and its legitimate causes, are of comparatively rare occurrence. If, then, we have such an almost universal evil to deal with as septicemia, and if that septicemia is, in thirty-seven cases out of thirty-nine, clearly traceable to the poisonous fluids effused in the peritoneal cavity, is it not self-evident that the indication, both of prevention and cure, is to draw off these poisonous fluids in the speediest and most direct way possible?"

The author awards to Dr. E. R. Peaslee the merit of having first recommended and practiced washing out the peritoneum with disinfectants, so early as 1855.

"To him (Dr. Peaslee) we owe the principle of intra-peritoneal medication. I claim only to be his coadjutor. But the rule that he would establish for exceptional cases I wish to make applicable to all alike; and instead of washing out the peritoneal cavity at the top, I propose to open it at the bottom, as he did in 1855, and let the fluids run out spontaneously and continuously. The natural outlet of the peritoneal cavity is through the Douglas *cul-de-sac.* If the drainage through this *cul-de-sac* be made promptly and thoroughly, there is every reason to believe that pyemic collections, such as happened to Mr. Spencer Wells, could never take place; and that septicemic poisoning would in a great measure be prevented."

Dr. Sims proposes, in brief, to puncture the *cul-de-sac* of the vagina behind the cervix uteri, and to pass a tube of some sort into the peritoneal cavity to drain off any effusion that may take place in said cavity. He proposes to do this in every case of ovariotomy, whether there are adhesions or not. If in three or four days we see that there is no necessity for this precautionary step in the operation, we have only to remove the tube, and in twenty-four hours the little puncture closes up spontaneously. The puncture adds nothing to the danger of ovariotomy, cannot possibly do the least harm, and may be the means of saving life.

### LATENT GONORRHŒA IN THE FEMALE.

Dr. E. Noegerath, of New York, recently published in Germany a small work under the above title, which has received a great deal of attention.

The theory of Dr. Noegerath is so startling, and so important in the physical and moral issues which are involved in its acceptance, that it demands the earnest and careful consideration of the medical profession.

The author concludes with these propositions:

"1. As a rule, gonorrhœa, both in the male and female, persists during the lifetime of the individual, in spite of apparent cure.

"2. There exists, therefore, a latent gonorrhœa in the male as well as the female sex.

"3. This latent disease, both in the male and female, may cause either a latent or an acute gonorrhœa in a previously healthy person.

"4. A latent gonorrhœa manifests itself in time as acute, chronic or recurrent peri-metritis, or ovaritis, or as a catarrhal affection of the individual parts of the mucous membrane of the genital tract.

"5. The wives of those men who, at any period of their lives, have had a gonorrhœa, remain, as a rule, sterile.

"6. Those who may become pregnant, either abort or bear only one child. In exceptional cases three or four children may be produced.

"7. A fungous can be developed from the vaginal secretions of women who are affected with a latent gonorrhœa, which is completely analogous to that obtained from the product of an acute attack of gonorrhœa in the male."

Dr. Noegerath estimates that eight hundred out of every one thousand men have suffered at some period of time during their life from gonorrhœa, and that these eight-tenths of our male population are "doomed beyond hope; no life-long repentance, no rigorous asceticism, no known method of treatment, no assurance derived from years of continued health and apparent freedom from disease, can save them, for the consequences of their sin will inevitably be visited—not on their children, for in the majority of cases paternity is denied them, but—on her whom they hold nearest and dearest, for *infection* and *sterility* must be her lot."

At a meeting of the Obstetrical Society of Edinburgh, held April 23, 1873, this subject was introduced for discussion by Dr. Angus Macdonald.

Dr. Macdonald believed that Dr. Noegerath had hold of a great idea, and one which possessed a large amount of evidence in its favor, and that although he regarded his assumptions as extravagant, they deserved the careful attention of gynæcologists.

Dr. Matthews Duncan coincided to some extent in the deductions of Dr. Noegerath, but he also thought that Dr. N. had gone too far in his conclusions.

Drs. Keiller and Young, however, affirmed that their experience did not support the views of Dr. Noegerath, and stated that they had often treated married men for gonorrhœa, and their wives had never complained of suffering from the symptoms alleged to be produced by it, and that they had never noticed any falling off in their fertility.

DILATATION OF THE FEMALE URETHRA.

Dr. Oscar H. Allis reports, in the November number of the *Obstetrical Journal of Great Britain and Ireland*, two cases in which he had successfully dilated the female urethra for the removal of two large calculi (the circumference of the largest being three inches and a half) in one case, and in the other of a polypus attached to a roughened, thickened portion of the bladder, at a point corresponding to the right side of the pubes. In both cases the dilatation was effected in about fifteen minutes, under ether, and in neither of them was there left any incontinence of urine.

